# Leisure Screen Time and the Risk of Six Neurodevelopmental Disorders: A Two‐Sample Mendelian Randomization Study

**DOI:** 10.1002/brb3.70884

**Published:** 2025-09-21

**Authors:** Chen Cai, Qimei Ran, Ming Lu, Chao Song, Zhongquan Jiang

**Affiliations:** ^1^ Hangzhou Linping Women & Children Health Hospital Hangzhou China; ^2^ Department of Developmental and Behavioral Pediatrics National Clinical Research Centre for Child Health Children's Hospital Zhejiang University School of Medicine Hangzhou China; ^3^ School of Public Health Lanzhou University Lanzhou China

**Keywords:** leisure screen time, Mendelian randomization, neurodevelopmental disorders

## Abstract

**Background:**

Neurodevelopmental disorders (NDDs)—including autism spectrum disorder, attention deficit hyperactivity disorder (ADHD), intellectual disability, learning disability, speech disorder, and Tourette disorder—impact brain development and impair social, learning, and occupational functioning. We performed a Mendelian randomization (MR) analysis using summary data from global genome‐wide association studies to investigate the potential causal relationship between leisure screen time (LST) and NDDs risk.

**Methods:**

Our dataset comprised 703,901 participants of European ancestry from 51 studies, with 256,725 individuals in the LST‐valid sample. We investigated causal associations with six types of NDDs using five MR methods: inverse‐variance weighted (IVW), MR Egger, weighted median, simple mode, and weighted mode. IVW was the primary method due to its efficiency and precision. Heterogeneity and horizontal pleiotropy were assessed using IVW and MR Egger, while the other methods served as sensitivity analyses to confirm robustness.

**Results:**

The IVW method revealed that each standard deviation increase in LST was associated with a reduced risk of ADHD (OR = 0.68; 95% CI: 0.52–0.89) and an elevated risk of intellectual disability (OR = 1.66; 95% CI: 1.26–2.18). These associations were consistent with the weighted median analysis (ADHD: OR = 0.68; 95% CI: 0.47–0.98; intellectual disability: OR = 1.51; 95% CI: 1.06–2.14).

**Conclusions:**

Our findings suggest that genetic predisposition to increased LST is causally associated with a lower risk of ADHD but a higher risk of intellectual disability, with no evidence for a causal relatawdionship with the other four NDDs. Larger or longitudinal studies are needed for further validation.

## Graphical abstract

Leisure Screen Time and Neurodevelopmental Disorders: A Genetic Perspective

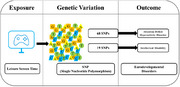

## 1 Introduction

Neurodevelopmental disorders (NDDs), including autism spectrum disorder (ASD), attention deficit hyperactivity disorder (ADHD), intellectual disability (ID), learning disability (LD), speech disorder (SD), and Tourette disorder (TD), are a group of conditions that affect brain development and function, leading to impairments in social, learning, or occupational abilities (Kalin 2020; Niemi et al. 2018; Parenti et al. 2020; Battle 2013). These disorders typically manifest early in development and are characterized by delays in motor, cognitive, and emotional milestones (Battle 2013). Evidence suggests that NDDs in children are a global issue, affecting approximately 3% of children worldwide (Gilissen et al. 2014). The prevalence of NDDs has been steadily increasing across the globe (Francés et al. 2022). The high prevalence of these noncommunicable diseases not only compromises children's health and social functioning but also imposes significant economic burdens on families (Peñuelas‐Calvo et al. 2021; Lopez et al. 2019). Currently, the underlying causes and mechanisms of NDDs remain poorly understood. Research indicates that genetic factors (Parenti et al. 2020; L. Wang, Owusu‐Hammond, et al. 2023), environmental influences (Thapar et al. 2013; Sánchez et al. 2024), and gut microbiota (Kim et al. 2022; Q. Wang, Yang, et al. 2023) may play a role in their development. Additionally, the severity of NDDs can be exacerbated by comorbid conditions (Khachadourian et al. 2023; Set and Warner 2021; Thapar and Cooper 2016) or psychological stress (Lautarescu et al. 2020; Bonis 2016). Given the lack of a clear understanding of the pathogenesis and the absence of effective treatments, gaining a comprehensive understanding of the risk factors associated with NDDs is essential for advancing research into their mechanisms and potential interventions.

Physical activity has a significant impact on neurodevelopment (Peng et al. 2022; Álvarez‐Bueno et al. 2018), as well as on various chronic diseases such as cardiovascular disease (Perry et al. 2023; Elagizi et al. 2020), diabetes (Perry et al. 2023; Aune et al. 2015; Kanaley et al. 2022), and cancer (McTiernan et al. 2019; Garcia et al. 2023). The increase in sedentary behavior, coupled with a decline in physical activity levels, poses a comparable threat to public health. Studies have shown that prolonged sedentary behaviors, particularly leisure screen time (LST), are associated with an increased risk of certain cancers and overall mortality (Hermelink et al. 2022; Kerr et al. 2017). Research has demonstrated that genetic loci associated with LST are enriched in genes whose expression in skeletal muscle is modulated by resistance training (Z. Wang, Emmerich, et al. 2022). However, there is very limited evidence associating LST—one of the primary sedentary activities—with an increased risk of NDDs. Furthermore, residual confounding factors and/or reverse causality limit the ability of observational studies to draw definitive causal conclusions.

The Mendelian randomization (MR) approach utilizes genetic variations as instrumental variables (IVs) to replace traditional risk factors in analysis (Burgess and Thompson 2017; Sekula et al. 2016; Emdin et al. 2017). Since genetic variations are randomly assigned during meiosis, this method is less susceptible to confounding factors that may bias observational studies. In the present study, we conducted an MR analysis using summary data from global genome‐wide association studies (GWAS) to better understand the potential causal relationship between LST and the risk of NDDs. This approach has been successfully applied in various medical fields, including investigating risk factors for deep vein thrombosis (Tan, Liu, et al. 2021), exploring causal relationships in mental health disorders (Liu et al. 2021), and examining cardiovascular disease mechanisms (Gao et al. 2022). In the present study, we conducted an MR analysis using summary data from GWAS to better understand the potential causal relationship between LST and the risk of NDDs.

## 2 Method

### 2.1 Study Design

**FIGURE 1:**
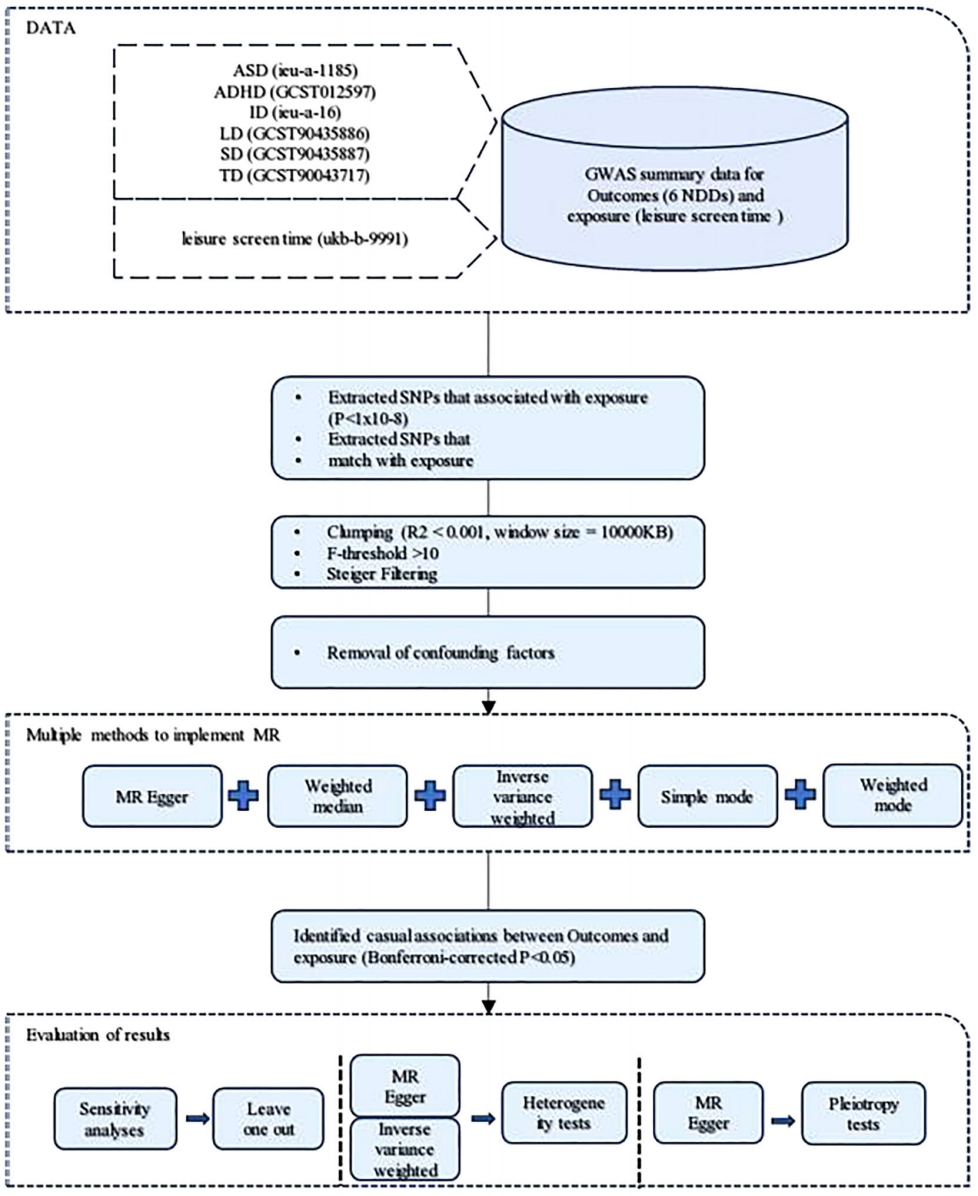
Overall workflow.

The overall workflow of this study is illustrated in Figure 1. In this research, LST was considered the exposure, while six types of NDDs served as the outcomes. Single‐nucleotide polymorphisms (SNPs) significantly associated with the exposure were used as IVs. For a robust MR study, each IV must meet the following criteria: it should be strongly associated with the exposure, independent of all other IVs and potential confounders, and influence the outcome only through the exposure. These principles were rigorously followed throughout the study. We conducted a comprehensive set of downstream analyses to address potential biases that could compromise the reliability of our findings. Specifically, we estimated the *F*‐statistic and performed Steiger testing to ensure the validity of the IVs. Horizontal pleiotropy and outliers were detected and corrected using Egger regression and Mendelian Randomization Pleiotropy Residual and Outliers (MR‐PRESSO). Leave‐one‐out (LOO) analysis was employed to assess the influence of key IVs, and reverse MR analysis was conducted to evaluate the potential for reverse causality.

### 2.2 Exposure and Outcome Data

GWAS data for LST were obtained from a publicly accessible web server (https://www.ebi.ac.uk/gwas/studies/). This dataset included individual‐level data from 703,901 participants of European ancestry across 51 studies, with 256,725 individuals included in the LST‐related valid sample. Outcome data included six types of NDDs. However, due to the lack of corresponding GWAS data for ID, intelligence scores from European populations were used as a proxy. Specifically, we selected childhood intelligence data under the Education subcategory from European populations as the proxy measure. Throughout the data analysis process, we employed continuous variables for MR. The GWAS data for the other NDDs were as follows; see Table 1: (1) ASD: GWAS with ID ieu‐a‐1185, European sample size of 46,351, including 18,382 cases; (2) ADHD: GWAS with ID GCST012597, European sample size of 21,191, including 4945 cases; (3) LD: GWAS with ID GCST90435886, European sample size of 408,542, including 164 cases; (4) SD: GWAS with ID GCST90435887, European sample size of 408,467, including 89 cases; and (5) TD: GWAS with ID GCST90043717, European sample size of 456,348, including 310 cases.

**TABLE 1:** Details of studies and datasets used for analyses.

Exposure/outcomes	ID	Year	Number of cases	Number of controls	Sample size	PubMed ID	Consortium
LST	GCST90104339 (Z. Wang, Emmerich, et al. 2022)	2022	NA	NA	526,725	36071172	European
ASD	ieu‐a‐1185 (GWAS 2017)	2017	18,382	27,969	46,351	—	European
ADHD	GCST012597 (Martin et al. 2018)	2018	4945	16,246	21,191	29325848	European
ID	ieu‐a‐16 (GWAS 2014)	2014	NA	NA	12,441	—	European
LD	GCST90435886 (Zhou et al. 2018)	2018	164	408,378	408,542	30104761	European
SD	GCST90435887 (Zhou et al. 2018)	2018	89	408,378	408,467	30104761	European
TD	GCST90043717 (Jiang et al. 2021)	2021	310	456,038	456,348	34737426	European

### 2.3 Two‐Sample MR (2SMR) and Causal Effect Assessment

This study required a strong association between the exposure and the outcomes, defined as SNPs significantly associated with traits (*p* ≤ 5×10^−8^), Hardy–Weinberg disequilibrium (*p* ≤ 0.001), or linkage disequilibrium (*r* ^2^ ≤ 0.001). Exposure and outcome data were harmonized such that the effect alleles reflected the alleles associated with the exposure. For palindromic SNPs (i.e., A/T or G/C), allele frequency information was used to resolve strand ambiguity. SNPs without *p*‐values, beta coefficients, or standard errors (SE) of beta coefficients were excluded from the GWAS catalog.

Using the results from GWAS, we applied a 2SMR approach, where SNP‐exposure effects and SNP‐outcome effects originated from distinct studies. This enabled the estimation of the causal effect of the exposure on the outcomes.

The causal associations were investigated using traditional MR methods, including inverse‐variance weighted (IVW), MR Egger, weighted median, simple mode, and weighted mode methods. The IVW method was considered the primary analytical approach due to its efficiency and precision under the assumption of valid instruments or balanced pleiotropy. Heterogeneity tests were conducted during MR analyses using both IVW and MR Egger methods. Horizontal pleiotropy, which can distort the association between the exposure and the outcome, may lead to inflated or deflated effect estimates when using the IVW method. To formally assess unbalanced horizontal pleiotropy, we adopted the MR Egger method, which provides robust MR estimates while accounting for pleiotropy. In this study, causal relationships were considered “strict” and could only be confirmed when associations observed using the IVW method passed significance thresholds.

### 2.4 Statistical Analysis

*p*‐values were two‐sided, and associations were considered statistically significant when *p* < 0.05. Bonferroni correction was applied to account for multiple testing, adjusting the *p*‐values accordingly. All statistical analyses were conducted using R version 4.2.1 (http://www.r‐project.org).

## 3 Results

### 3.1 Causal Associations

The IVW method showed that for each standard deviation (hour) increase in LST, the odds ratio (OR) for ADHD was 0.68 (95% confidence interval (CI): 0.52, 0.89), while the OR for intelligence development was 1.66 (95% CI: 1.26, 2.18). These results were consistent with the weighted median methods (ADHD: OR = 0.68 [95% CI: 0.47, 0.98], *p* < 0.05; ID: OR = 1.51 [95% CI: 1.06, 2.14], *p* < 0.05). Thus, we have strong evidence to confirm that genetically predicted LST is a protective factor for ADHD but a risk factor for intelligence development. This suggests that LST exerts a causal influence on NDDs to some extent; see Table 2.

**TABLE 2:** Causal associations between genetically determined exposure and outcome.

Outcome	Method	Exposure—LST					
		SNPs	Beta	SE	*p*‐value	OR	95% CI
ASD	MR Egger	72	0.239	0.479	0.619	1.27	0.50, 3.25
	Weighted median	72	0.166	0.106	0.117	1.18	0.96, 1.45
	Inverse variance weighted	72	−0.045	0.098	0.643	0.96	0.79, 1.16
ADHD	MR Egger	68	−0.624	0.685	0.365	0.54	0.14, 2.05
	Weighted median	68	−0.386	0.185	0.037	0.68	0.47, 0.98
	Inverse variance weighted	68	−0.383	0.136	0.005	0.68	0.52, 0.89
ID	MR Egger	19	−0.192	0.800	0.814	0.83	0.17, 3.96
	Weighted median	19	0.412	0.179	0.021	1.51	1.06, 2.14
	Inverse variance weighted	19	0.505	0.140	< 0.001	1.66	1.26, 2.18
LD	MR Egger	71	−1.755	2.637	0.508	0.17	0, 30.370
	Weighted median	71	0.250	0.808	0.757	1.28	0.26, 6.26
	Inverse variance weighted	71	0.083	0.555	0.880	1.09	0.37, 3.23
SD	MR Egger	71	−1.918	3.653	0.601	0.15	0.00, > 100.00
	Weighted median	71	0.236	1.077	0.826	1.27	0.15, 10.46
	Inverse variance weighted	71	−0.396	0.765	0.605	0.67	0.15, 3.01
TS	MR Egger	71	2.777	1.939	0.157	16.07	0.36, > 100.00
	Weighted median	71	−0.188	0.555	0.735	0.83	0.28, 2.46
	Inverse variance weighted	71	−0.209	0.402	0.603	0.81	0.37, 1.78

**TABLE 3:** Heterogeneity test and horizontal pleiotropy results in six NDDS.

Outcome	Index	Egger intercept	SE	*p*‐value
ASD	Heterogeneity test	143.65	71.00	< 0.001
	Horizontal pleiotropy	−0.01	0.01	0.545
ADHD	Heterogeneity test	77.86	67.00	0.171
	Horizontal pleiotropy	0.01	0.02	0.720
ID	Heterogeneity test	22.20	18.00	0.223
	Horizontal pleiotropy	0.02	0.02	0.388
LD	Heterogeneity test	68.86	70.00	0.516
	Horizontal pleiotropy	0.05	0.07	0.478
SD	Heterogeneity test	72.19	70.00	0.405
	Horizontal pleiotropy	0.04	0.10	0.671
TS	Heterogeneity test	52.19	70.00	0.945
	Horizontal pleiotropy	−0.08	0.05	0.120

No significant effects of genetically predicted LST were observed for ASD, LD, SD, or TD (*p* ≥ 0.05). Notably, the results for ADHD and ID were not significant in the MR Egger analysis.

Both IVW and MR Egger estimations showed no evidence of heterogeneity in the causal effects of LST on ADHD across 68 SNPs (*p* ≥ 0.05). Similarly, no evidence of horizontal pleiotropy was found in the MR Egger regression (*p* ≥ 0.05). For intelligence development, no heterogeneity was detected across the 19 SNPs (*p* ≥ 0.05), nor was there evidence of horizontal pleiotropy (*p* ≥ 0.05); see Table 3.

## 4 Discussion

Using comprehensive genetic data from nearly 1.5 million individuals, this study demonstrated the causal relationship between LST and six different NDDs. The main findings suggest that genetically predicted LST reduces the risk of ADHD while increasing the risk of impaired intelligence development.

Currently, there is no gold standard for MR analysis methods. Following the “no free lunch” principle (Adam et al. 2019), we applied multiple MR approaches (MR Egger, weighted median, and IVW methods) to assess the robustness of the causal relationship between exposure (LST) and outcomes (NDDs). This allowed us to evaluate the exposure–outcome relationships more comprehensively.

In recent years, as the prevalence of NDDs among children has risen (Sánchez et al. 2024), researchers have conducted extensive studies on the effects of screen exposure on NDD‐related diseases. Most cross‐sectional studies have shown a significant relationship between increased screen exposure and ADHD symptoms, as well as a connection to later gaming addiction (Engelhard and Kollins 2019; Marco et al. 2009). Some longitudinal studies have also demonstrated a significant association between digital media use and ADHD symptoms, indicating a positive correlation between screen time and subsequent ADHD or attention symptoms (Ra et al. 2018; Sibley and Coxe 2018). However, other studies have found no such relationship.

For example, Niiranen et al. (2024) investigated the relationship between screen time at 18 months of age and ADHD symptoms at 5 years, defining excessive screen time as more than 45 min per day. Poulain et al. (2019) surveyed children aged 10–17 and found significant associations between computer/internet use and outcomes such as peer relationships, well‐being, and quality of life, but no association with ADHD symptoms. These positive and negative results are not entirely contradictory to our findings. Our study specifically focused on LST, while many of the aforementioned studies concentrated on digital media addiction. LST differs from the overstimulation, behavioral imitation, and disrupted sleep rhythms often caused by digital media addiction. Moderate LST may improve mood and attention, while certain open‐world games can stimulate creativity, allowing children to express themselves in a safe environment and enhance self‐regulation skills. Therefore, compared to prolonged, highly stimulating digital media use, moderate LST may not negatively impact attention and might even provide beneficial stimulation.

In contrast to ADHD, genetically predicted LST increases the risk of impaired intelligence development. While our study suggests that LST is a causal factor in reduced intelligence development, the causal relationship may vary depending on the type of LST. It is difficult to draw a direct conclusion about whether LST generally impairs children's intellectual development. We also attempted reverse MR analyses to assess whether NDDs causally influence LST, but due to insufficient valid genetic instruments for ADHD and ID, these analyses were not feasible. Future studies with stronger instruments are needed to clarify potential bidirectional relationships.

Some studies have shown that activities such as watching TV and online videos, socializing via social media, texting, video chatting, and gaming have varying impacts on intelligence. Gaming has been found to have a positive impact on changes in intelligence (Zhao et al. 2022). The Flynn effect (Trahan et al. 2014)—the gradual increase in global IQ scores over the decades—can partly be explained by the positive cognitive effects of watching videos and playing video games. Innovations in electronic and information technology (e.g., films, television, video games, computers, the internet) have created an iterative process between technology users and designers. Generations growing up with increasingly complex digital media have developed higher cognitive demands, contributing to advancements in technology. However, some studies have also shown that excessive LST exposure can negatively impact cognition and intelligence development (Zhao et al. 2022; John et al. 2021; Vohr et al. 2021). For example, a recent MR study highlighted the causal effects of screen time on childhood intelligence, emphasizing the mediating role of intracranial volume (Feng et al. 2025). Given the diversity of cultural backgrounds, genetic profiles, and economic environments, it is challenging to disentangle the dose–response relationship between LST and intelligence development.

The clinical implications and mechanisms of LST's effects on NDDs present a complex paradigm. For ADHD, moderate LST may offer protective effects through enhanced cognitive control networks and structured attention regulation, distinguishing it from the adverse impacts of digital media addiction. Regarding intelligence development, LST's influence appears to operate through dual pathways: While certain forms of digital engagement may enhance neural plasticity and cognitive development, excessive LST might impair intelligence development by reducing essential real‐world learning experiences. These findings suggest that LST's impact on neurodevelopment involves multiple neurobiological mechanisms, including changes in synaptic plasticity and cognitive processing patterns. Understanding these relationships is crucial for developing evidence‐based guidelines for optimal LST exposure, considering individual variations in genetic susceptibility and developmental stages.

This study has several limitations: (1) If the exposure represents a composite trait with multiple subtypes, it is possible that the observed effects are primarily influenced by a single subtype, leaving the causal relationships identified uncertain and in need of confirmation through future randomized controlled trials (RCTs). (2) The limited information from GWAS summary data led us to assume a linear relationship between traits and diseases, though the true association may follow a “J‐shaped” or “U‐shaped” curve. (3) Given the significant genetic differences between Eastern and Western populations, as evidenced by the distinct mutation patterns observed between Chinese and European populations (Tan, Yan, et al. 2021), our findings based on European ancestry populations may not be directly applicable to other ethnic groups and require further validation in diverse populations. (4) Due to restrictions in variables and data availability, the influence of other unmeasured factors and pathways cannot be excluded.

## 5 Conclusion

In conclusion, using 2SMR analysis, the results indicate that genetic exposure leading to increased LST risk is associated with a reduced risk of ADHD but an increased risk of intellectual development. However, the current data do not support a causal relationship between genetic exposure to increased LST risk and the other four NDDs. Further large‐scale or longitudinal studies are needed to validate these findings.

## Author Contributions

Chen Cai and Qimei Ran designed the statistical method, performed lysis, and drafted the manuscript. Ming Lu and Zhongquan Jiang conceived the idea, devised, and supervised the study. Chao Song conceived the idea, supervised, and acquired funding for the study. All authors revised and approved the final manuscript.

## Disclosure

The authors have nothing to report.

## Conflicts of Interest

The authors declare no conflicts of interest.

## Peer Review

The peer review history for this article is available at https://publons.com/publon/10.1002/brb3.70884.

## Data Availability

The data that support the findings of this study are available on request from the corresponding author. The data are not publicly available due to privacy or ethical restrictions.
